# Physical and Statistical Pattern of the Thiva (Greece) 2020–2022 Seismic Swarm

**DOI:** 10.3390/e27090979

**Published:** 2025-09-19

**Authors:** Filippos Vallianatos, Eirini Sardeli, Kyriaki Pavlou, Andreas Karakonstantis

**Affiliations:** 1 Section of Geophysics-Geothermics, Department of Geology and Geoenvironment, National and Kapodistrian University of Athens, 15784 Athens, Greece; eirsard@geol.uoa.gr (E.S.); kpavlou@geol.uoa.gr (K.P.); akarakon@geol.uoa.gr (A.K.); 2 Institute of Physics of Earth’s Interior and Geohazards, UNESCO Chair on Solid Earth Physics and Geohazards Risk Reduction, Hellenic Mediterranean University Research & Innovation Center, 73133 Chania, Greece

**Keywords:** Thiva swarm, clusters, seismicity, Tsallis entropy, afterslip, diffusion, DBSCAN

## Abstract

On 2 December 2020, an earthquake with a magnitude of *M*_w_ 4.5 occurred near the city of Thiva (Greece). The aftershock sequence, triggered by ruptures on or near the Kallithea fault, continued until January 2021. Seven months later, new seismic activity began a few kilometers west of the initial events, with the swarm displaying a general trend of spatiotemporal migration toward the east–southeast until the middle of 2022. In order to understand the physical and statistical pattern of the swarm, the seismicity was relocated using HypoDD, and the magnitude of completeness was determined using the frequency–magnitude distribution. In order to define the existence of spatiotemporal seismicity clusters in an objective way, the DBSCAN clustering algorithm was applied to the 2020–2022 Thiva earthquake sequence. The extracted clusters permit the analysis of the spatiotemporal scaling properties of the main clusters using the Non-Extensive Statistical Physics (NESP) approach, providing detailed insights into the nature of the long-term correlation of the seismic swarm. The statistical pattern observed aligns with a *Q*-exponential distribution, with *q_D_* values ranging from 0.7 to 0.8 and *q_T_* values from 1.44 to 1.50. Furthermore, the frequency–magnitude distributions were analyzed using the fragment–asperity model proposed within the NESP framework, providing the non-additive entropic parameter (*q_M_*). The results suggest that the statistical characteristics of earthquake clusters can be effectively interpreted using NESP, highlighting the complexity and non-additive nature of the spatiotemporal evolution of seismicity. In addition, the analysis of the properties of the seismicity clusters extracted using the DBSCAN algorithm permits the suggestion of possible physical mechanisms that drive the evolution of the two main and larger clusters. For the cluster that activated first and is located in the west–northwest part, an afterslip mechanism activated after the 2 September 2021, *M* 4.0 events seems to predominately control its evolution, while for the second activated cluster located in the east–southeast part, a normal diffusion mechanism is proposed to describe its migration pattern. Concluding, we can state that in the present work the application of the DBSCAN algorithm to recognize the existence of any possible spatiotemporal clustering of seismicity could be helping to provide detailed insight into the statistical and physical patterns in earthquake swarms.

## 1. Introduction

**Figure 1:**
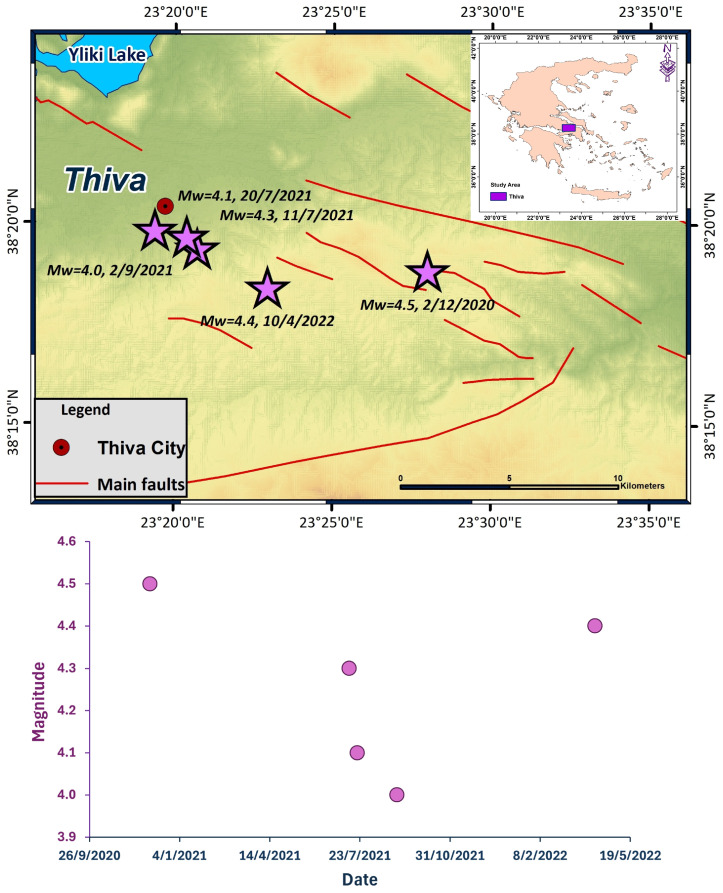
(**Up**) The spatial distribution of major seismic events (purple stars)in Thiva from 25 November 2020 to 1 July 2022. Faults are marked with red lines [7]. (**Down**) The temporal evolution of the major seismic events.

The area of Thiva is located between the Gulf of Corinth and Evia (central Greece), with normal faults oriented in WNW-trending in the eastern part and ENE-trending in the western part [1,2]. Historical records show that this area experienced significant seismic activity in the 19th century. More specifically, a devastating earthquake of magnitude *M* 7.0 occurred in the Thiva region in 1853 [3], which affected the area between Thiva and Yliki Lake. The next significant seismic event was on 23 May 1893, with a magnitude of *M* 6.0 [4]. In addition, the earthquake of 17 October 1914, with a magnitude of *M*_s_ 6.2, caused severe damage in the city of Thiva and nearby villages [5]. Until 2020, Thiva had not witnessed any significant seismic activity in recent years. However, on 2 December 2020 at 10:54:56 UTC, an earthquake of magnitude 4.5 occurred east of the city of Thiva (Figure 1), initiating a notable series of earthquakes that persisted until June 2022. The 2020 seismic events were concentrated mainly in the eastern part of the study area, occurring along a system of normal faults. The mainshock and the aftershocks were caused by ruptures on or near the Kallithea fault, which dips SW [6,7]. Subsequently, heightened seismic activity commenced on 10 June 2021, with significant events on 11 July 2021, 20 July 2021, and 2 September 2021, of magnitudes *M*_w_ 4.3, 4.1, and 4.0, respectively. The seismic activity on 10 July 2021, started at the western part of the swarm, and the following seismic events demonstrated a general trend for spatiotemporal migration towards east–southeast. The final significant event occurred on 10 April 2022, with a magnitude of *M*_w_ 4.4.

In the present study, a clustering algorithm is initially applied to the 2020–2022 earthquake sequence of Thiva (Greece) to identify the existence of spatiotemporal clusters and to support the validation of underlying mechanisms that drive seismicity in each cluster. Particularly, the DBSCAN algorithm [8] is utilized, which detects clusters in extensive databases by identifying regions of high point density while treating isolated or sparsely populated points as noise. Nevertheless, a different approach to the DBSCAN algorithm is presented, which intends to identify high-density regions in large datasets while treating isolated events as noise. Our modified approach further allows the recognition of clusters that are correlated in both space and time. Before the clustering algorithm was applied, the hypocenters were relocated using the HypoDD code [9] for a more accurate insight into the seismicity distribution. Furthermore, the frequency–magnitude distribution in terms of Gutenberg–Richter is used to calculate the magnitude of completeness (*M*_c_). The clustering algorithm, including seismic events equal to and above the *M*_c_, is applied to the catalog to ensure more accurate results. Subsequently, we selected the two largest clusters out of the five for further analysis. Afterwards, the spatiotemporal scaling properties of the two largest clusters were analyzed, using the Non-Extensive Statistical Physics (NESP) framework. This innovative approach, as developed by Tsallis [10] and extensively applied in geosciences [11,12,13,14] and references therein, aims to provide more detailed insights into the nature of the long-term correlation of the earthquake generation process. Hence, the fragment–asperity model, along with the distribution of inter-event times and distances between the successive events, is analyzed within the framework of Non-Extensive Statistical Physics (NESP), providing the non-additive entropic parameters (*q_T_*, *q_D_*, and *q_M_*) [11]. In addition, possible physical mechanisms that drive the evolution of the two main and larger clusters are suggested. For the first cluster, an afterslip mechanism activated after the 2 September 2021, *M* 4.0 events seems to control its evolution, while for the second cluster, a normal diffusion mechanism is proposed to describe its migration pattern.

## 2. Seismological Data

### 2.1. Relocation Using HypoDD

The earthquake catalog for the 2020–2022 Thiva earthquake swarm was extracted from the Seismological Laboratory of the National and Kapodistrian University of Athens (SL-NKUA) for the period between 25 November 2020 and 13 June 2022. During this period, the catalog includes 4711 events with depths ranging from 0 to 30 km.

The above catalog was used in the relocation procedure. In the first stage of the sequence analysis, hypocenters were located in near real-time using a single-event program, such as HypoInverse [15], along with a custom regional 1D velocity model [16]. The final catalog was processed using HypoDD, a double-difference algorithm [17] that minimizes the double-difference residuals for pairs of earthquakes at each station by weighted least squares, employing the conjugate gradients method (LSQR, [18]).

**Figure 2:**
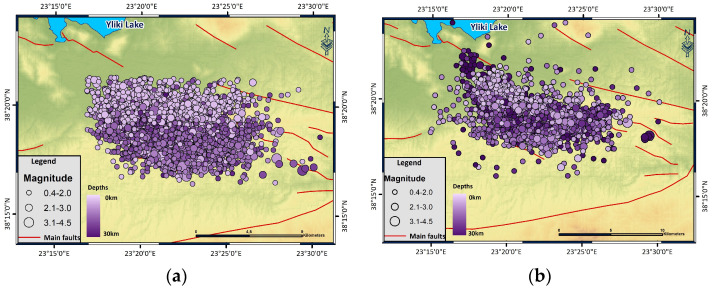
(**a**) Initial location of the 2020–2022 Thiva earthquake sequence in comparison with (**b**) the relocated events using HypoDD of the 2020–2022 Thiva earthquake sequence that occurred between 25 November 2020 and 13 June 2022. Faults are marked with red lines [7].

In the Thiva area, 4182 out of 4711 events of the initial catalog were relocated using HypoDD. The relocated catalog refers to the period between 25 November 2020 and 13 June 2022, consisting of events with a depth distribution between 0 and 30 km and magnitudes of *M*_w_ 0–4.5 (Figure 2).

### 2.2. Catalogue Completeness

The well-known Gutenberg–Richter [19] scaling relation (G-R) indicates a power law behavior and is defined as follows:

(1) logN(>M)=a−bM

where $N\left(>M\right)$ represents the number of earthquakes with magnitude greater than *M*, and *α* and *b* are positive constants. The α value reflects the total seismicity rate and shows significant variations from one area to another. The parameter *b*, also known as the *b* value, is the slope of the frequency–magnitude distribution (FMD) and indicates the proportion of minor to major earthquakes. The *b* value in Equation (1) can be estimated by maximum likelihood using the equation [20,21,22] $b=\left(\frac{\log e}{\bar{M}-M_{min}}\right)$, where $\bar{M}$ is the observed mean magnitude and Mmin is the minimum magnitude of the given sample.

The magnitude of completeness, *M*_c_, is a crucial parameter in seismicity studies and is obtained after a correction of Mmin=Mc − ΔM/2, where Δ*Μ* is the binning width of the catalog (often set to 0.1) [20,22,23,24]. A correct estimation of the *b* value depends on *M*_c_. In this study, the magnitude of completeness *M*_c_ was estimated using a combination of the maximum curvature method and the goodness-of-fit test with 95% and 90% residuals [24]. The calculation was performed using the Zmap software package, version 7.0 [25].

**Figure 3:**
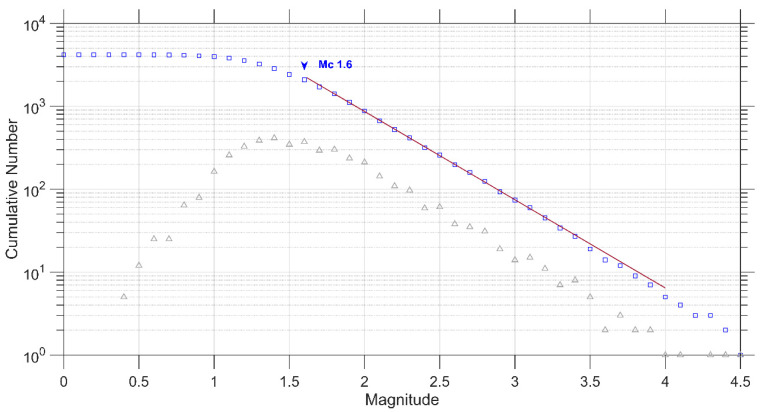
The frequency–magnitude distribution for the 2020–2022 Thiva Earthquake Swarm and the estimated *M*_c_ is plotted, showing both the cumulative (blue squares) and the discrete (gray triangles) frequency–magnitude distributions. The red solid line represents the G-R relation.

The FMD for the 2020–2022 Thiva Earthquake Swarm is depicted in Figure 3, along with the fitting according to the G-R scaling relation for the calculated model parameters. The estimated values are α = 4.885, *b*-value = 0.98 ± 0.02, and *M*_c_ = 1.6.

## 3. Analysis of Seismicity and Discussion

### 3.1. The DBSCAN Algorithm Results

DBSCAN (Density-Based Spatial Clustering of Application with Noise) [8] is recognized as one of the most powerful clustering methods, selected for its capability to analyze huge datasets effectively, and has a significant application in seismology [26,27,28]. The purpose of DBSCAN is to identify clusters of arbitrary shapes in extensive databases containing noise, using a density-based approach. The algorithm is based on the following input parameters: (a) the ε (epsilon value or eps), the distance (radius) around a given point, and (b) MinPoints (minimum number of samples or MinPts), the minimum number of points (including the point itself) that should be within the epsilon distance. These parameters are the same for all clusters.

The value of epsilon (ε) can be determined from the k-distance graph. This graph is constructed by plotting the distance to the kth nearest neighbor for each point in descending order [8]. The point on the graph where maximum curvature (elbow) is observed indicates the optimal value for epsilon. According to Ester et al. [8], the minimum number of points can be set to twice the number of dimensions, i.e., four points for 2D data, six points for 3D data, and eight points for 4D data.

In this study, the DBSCAN was utilized for the spatio-temporal clustering analysis of the 2020–2022 Thiva earthquake swarm. In addition to the three spatial dimensions (latitude, longitude, and depth), a fourth dimension, date–time, was added. The algorithm was applied to the relocated catalog of Thiva, which contains 2079 earthquake events with *M* ≥ *M*_c_ between 25 November 2020, and 13 June 2022.

**Figure 4:**
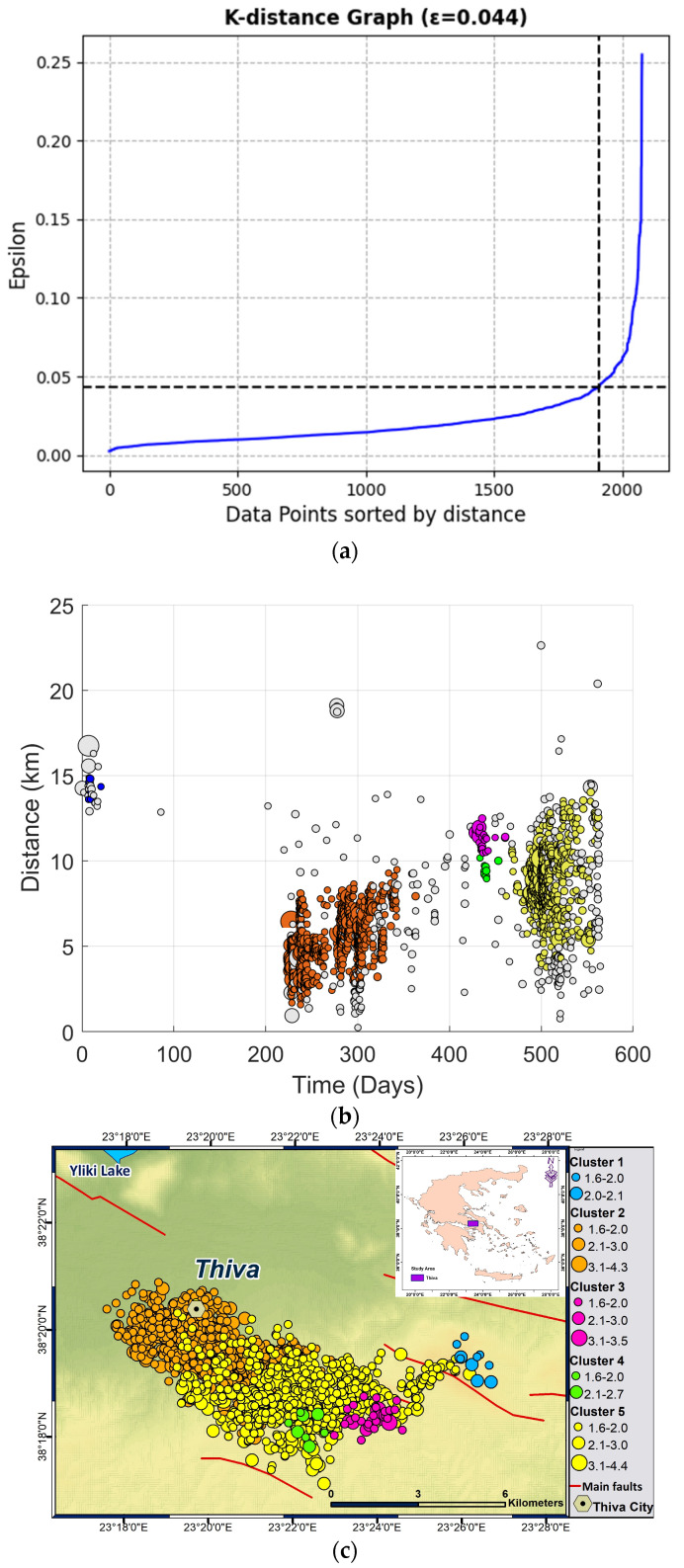

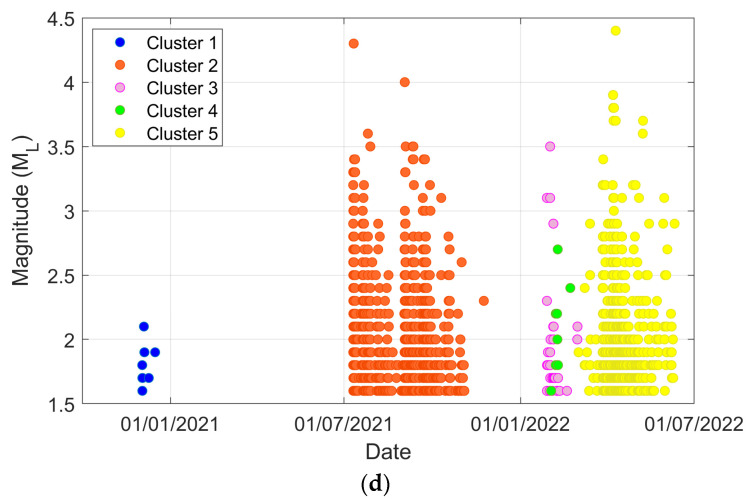
(**a**) The k-distance graph (k = 4 in our case) for the 2020–2022 Thiva earthquake sequence, with the threshold point indicated by the black dashed lines. (**b**) The distance–time plot showing the five clusters identified by the DBSCAN algorithm. The blue, orange, purple, green, and yellow dots correspond to clusters 1, 2, 3, 4, and 5, respectively. The gray dots represent the noise points. (**c**) Seismicity map of Thiva illustrating the identified earthquake clusters. The seismic events are categorized by size and color based on their magnitude and cluster number. (**d**) The magnitude–date distribution for the different events and the clusters observed.

The epsilon value was specified from the k-distance graph [8] (Figure 4a), with the optimal epsilon value for the Thiva earthquake catalog set at 0.044. Furthermore, the value of MinPts was set to 8, and a normalized Euclidean distance was applied to the spatial and temporal width of the four-dimensional data. The temporal outcome of the DBSCAN algorithm is illustrated in Figure 4b, showing the evolution of events with time, counting in days since 2 December 2020. Event distances in Figure 4b are calculated from an initial point at latitude 38.35 N and longitude 23.28 E, which is located at the western edge of the earthquake swarm. In Figure 4c the spatial distribution of clusters is presented, while in Figure 4d the magnitude–date distribution of the events, along with the cluster to which they belong, is demonstrated. The algorithm provided 5 clusters (see Table 1) and detected 366 noise points as a separate cluster, which were not part of any of the clusters due to being either too isolated or not densely connected to other points.

Among the five clusters recognized (see Table 1), two of them had a predominant importance since the majority of seismicity was located within these two clusters. Cluster 2 includes 1081 events, while Cluster 5 includes 644.

### 3.2. A Non-Extensive Statistical Physics Approach (NESP)

Earthquakes exhibit intricate phenomenology characterized by a fractal structure. These complex systems, with strongly correlated elements, typically exhibit power-law behavior. Consequently, the statistical mechanics description of such systems appears better represented by a generalized framework introduced by Tsallis [29,30] known as Non-Extensive Statistical Physics (NESP), which generalizes Boltzmann–Gibbs statistical mechanics.

Non-Extensive Statistical Physics (NESP) offers a more suitable approach for analyzing complex dynamic systems. It is based on a non-additive entropy, denoted as $S_{q}$ (commonly referred to as Tsallis entropy). This entropy is expressed through the following integral formulation:

(2) $$S_{q}=k_{B}\frac{1-\int_{0}^{\infty }p^{q}\left(X\right)dX}{q-1}$$

where $k_{B}$ represents Boltzmann’s constant, while the entropic index *q* characterizes the degree of non-additivity in the system. This is particularly relevant for complex systems that deviate from the additive nature of classical Boltzmann–Gibbs (BG) entropy. The given expression for $S_{q}$ applies to a continuous variable X, described by a probability distribution *p*(*X*), where 0 ≤ *p*(*X*) ≤ 1.

#### 3.2.1. Frequency–Magnitude Distribution of Earthquake Clusters in Terms of NESP

Sotolongo-Costa and Posadas [31], based on the fragment–asperity model, proposed a physical model for the earthquake frequency–size distribution. In [31] an energy-distribution function was introduced, and it was illustrated how the size distribution of fragments affects the energy distribution of earthquakes, using the non-extensive formalism (NESP), extracting the Gutenberg–Richter law as a particular case. Long-range interactions between all parts of existing fragments are expected due to the material’s severe fragmentation, implying the need for Non-Extensive Statistical Physics. According to Telesca [11], the following modified function that connects the cumulative number of earthquakes with the magnitude was established based on the Non-Extensive Statistical Physics theory:

(3) $$log\left(\frac{N(>M)}{N}\right)=\frac{2-q_{M}}{1-q_{M}}log\left(\frac{1-\left(\frac{1-q_{M}}{2-q_{M}}\right)\left(\frac{10^{M}}{A^{2/3}}\right)}{1-\left(\frac{1-q_{M}}{2-q_{M}}\right)\left(\frac{10^{Mc}}{A^{2/3}}\right)}\right)$$

where *M* is the earthquake magnitude, *M*_c_ is the completeness magnitude, and *A* is a parameter proportional to the volumetric energy density. In the fragment–asperity model, the non-extensive parameter *q_M_* indicates the degree of interactions within the system’s elements [14,31,32,33]. As a result, an increase in *q_M_* shows that the physical condition of a seismic region is moving away from equilibrium.

The normalized cumulative magnitude distribution of the Unified Cluster and the two main clusters (Cluster 2 and Cluster 5) is examined using the NESP model of Equation (3), for the entire range of magnitudes above a threshold equivalent to the completeness magnitude. The Unified Cluster involves the 1713 seismic events from the five DBSCAN clusters.

**Figure 5:**
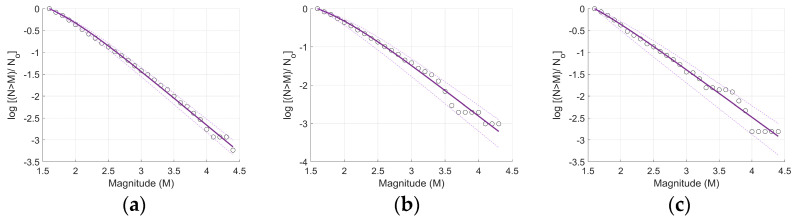
The frequency–magnitude distribution of the seismicity (circles) and its fitting (solid purple line) according to the fragment–asperity model for (**a**) Unified Cluster, (**b**) Cluster 2, and (**c**) Cluster 5 is presented. The 95% confidence intervals are plotted with dashed lines.

The model satisfactorily describes the observed magnitude distribution for each cluster. In Figure 5, the bold purple line represents the distribution function of Equation (2) for the estimated entropic parameter *q_M_*, while the other two dashed lines show the 95% confidence intervals. The estimated values are presented in Table 2.

#### 3.2.2. Spatiotemporal Scaling Properties of Earthquake Clusters in Terms of NESP

Seismicity is regarded as a point process in time, where the cumulative distribution *P*(>*T*) of the inter-event time *Ti = t(i + 1) − t(i)*, where *t(i)* is the time of occurrence of the *i*th event, and the cumulative distributions *P*(>*D*) of inter-event geodetic distance *D* estimated using the Vincenty inverse formula for ellipsoids between the foci of successive seismic events [34] play a crucial role for the spatiotemporal pattern characterization.

Using the Tsallis entropy, the cumulative distribution function (CDF) *P*(>*X*) is estimated using the *Q*-exponential distribution, defined for *q* < 2, where the constant *X* may express the inter-event times (*T*) or inter-event distances (*D*), respectively:

(4) $$P\left(>X\right)=\;{\left[1-(1-Q)\left(\frac{X}{X_{0}}\right)\right]}^{\frac{1}{1-Q}}$$

The *Q*-exponential distribution is derived through the maximization of non-additive entropy, $S_{q}$, by using the Lagrange multipliers method under relevant constraints [35]. To estimate the values of *q* and *X*_0_, q=2−(1/Q) and $X_{q}=X_{0}/Q$ are used [36]. For values of *Q* greater than 1, the *Q*-exponential distribution demonstrates asymptotic power-law behavior, while for *Q* equal to 1, it recovers the ordinary exponential expression.

The inverse of the *Q*-exponential function leads to the following *Q*-logarithmic function [35]:

(5) $${ln}_{Q}P\left(>X\right)=\frac{{P(>X)}^{1-Q}-1}{1-Q}$$

After determining the suitable *Q* value that best characterizes the distribution of *X*, the *Q*-logarithmic distribution displays linearity concerning *X*. We first fit the observed *P*(>*X*) using the *Q*-exponential distribution (Equation (4)) and then utilize the *Q*-logarithmic distribution (Equation (5)) as a goodness of linear fit between the observed data and the estimated *Q*-value (or *q*-value equivalently), as suggested by Equation (5).

**Figure 6:**
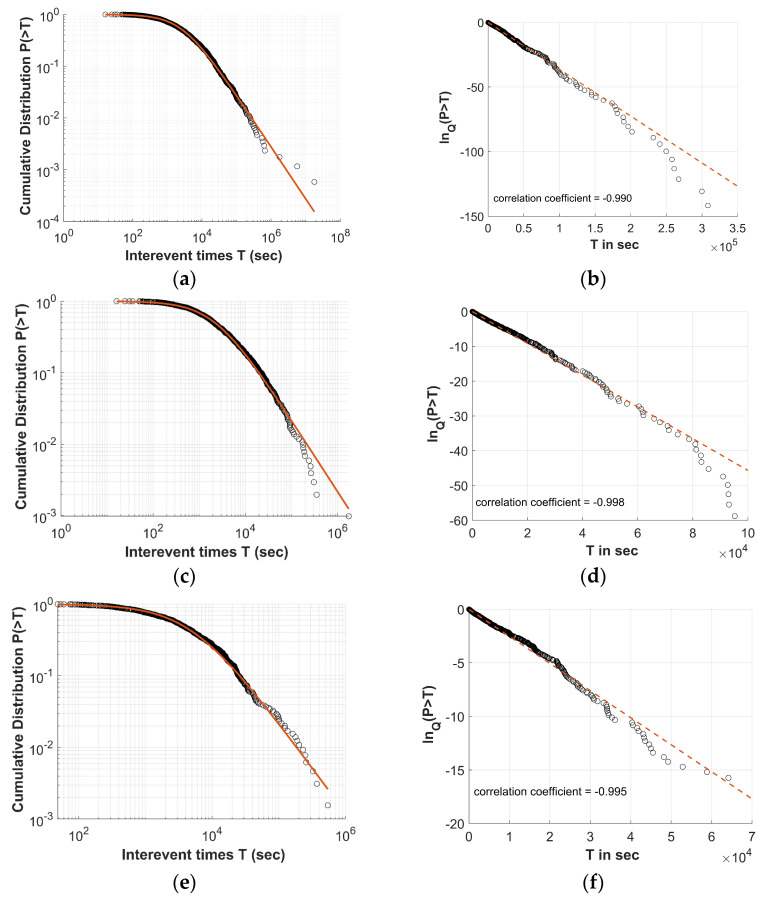
The left panels illustrate the log–log plot of the inter-event times cumulative distribution for the Thiva swarm, showing (**a**) Unified Cluster, (**c**) Cluster 2, and (**e**) Cluster 5. The solid brown line represents the fitting of the *Q*-exponential distribution for the q_T_ and *Tq* values. The right panels show the corresponding semi-*q*-log plot of the cumulative distribution of the inter-event times, illustrating (**b**) Unified Cluster, (**d**) Cluster 2, and (**f**) Cluster 5. The *Q*-logarithmic distributions exhibit a correlation coefficient, presented within the panels, while the dashed line is the *Q*-exponential fit.

For the Clusters 2 and 5 defined by the DBSCAN algorithm for the Thiva earthquake swarm, the cumulative distributions of the inter-event times *P*(>*T*) between the successive earthquakes are quite well described by the *Q*-exponential distribution, with an entropic parameter *q* greater than one. The corresponding *Q*-logarithmic distributions demonstrate linearity with high correlation coefficients, suggesting a strong linear fit between the model and the data (Figure 6) in a wide range of interevent time values.

**Figure 7:**
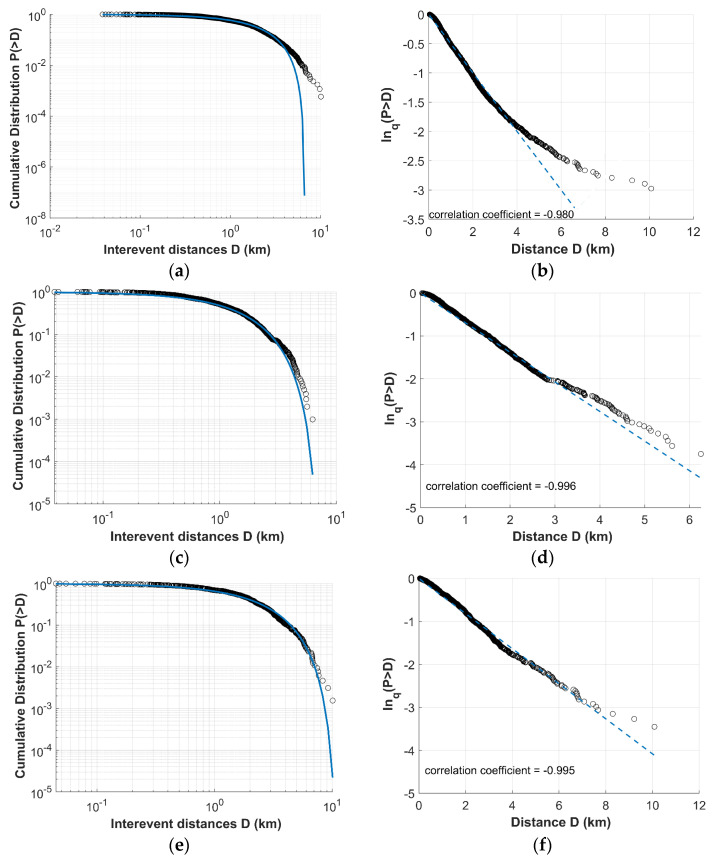
The left panels illustrate the log–log plot of the inter-event distances cumulative distribution for the Thiva swarm: (**a**) Unified Cluster, (**c**) Cluster 2, and (**e**) Cluster 5. The solid blue line indicates the fit of the *Q*-exponential distribution for the values of *q_D_* and *Dq*. The right panels depict the corresponding semi-*q*-log plot of the cumulative distribution of the inter-event distances: (**b**) Unified Cluster, (**d**) Cluster 2, and (**f**) Cluster 5. The *Q*-logarithmic distributions exhibit a correlation coefficient, presented within the panel, while the dashed line is the *Q*-exponential fit.

In Figure 7, the cumulative distributions of the inter-event distances *P*(>*D*) between the successive earthquakes are presented, showing a quite good fit with the *Q*-exponential distribution, characterized by an entropic parameter *q*_D_ < 1. As Equation (5) suggests, using the proper *Q* value, the *Q*-logarithmic function is linearly related with *D*. In Figure 7, the corresponding *Q*-logarithmic distributions demonstrate linearity with high correlation coefficients, indicating a strong goodness-of-fit between the model and the data. Table 3 presents the entropic parameters *q_T_* and *q_D_* values for Clusters 2 and 5 along with the *q* values for the dataset created by the merge of them. We note that the results are in agreement with those presented in [37], indicating that within each cluster of the earthquake swarm, the temporal evolution is described by low degrees of freedom, indicating a high level of organization, hierarchy, and non-additive characteristics.

### 3.3. Physical Model of Cluster’s Evolution

#### 3.3.1. Coulomb Stress Changes

In recent years, Coulomb stress changes induced by earthquakes have been extensively investigated to assess their role in aftershock sequence evolution and fault interaction [37,38,39,40,41,42,43,44]. These stress variations depend on the earthquake focal mechanism, fault geometry and slip, and the effective friction coefficient, providing a framework to evaluate how major events may trigger subsequent ruptures in the near field. Empirical studies have demonstrated a strong correlation between positive Coulomb stress changes and the spatial distribution of subsequent seismicity [37,45]. Specifically, changes in Coulomb failure stress (ΔCFS) can either advance or delay fault rupture, thereby influencing the timing and spatial evolution of seismic sequences [41,46,47]. Positive ΔCFS promotes failure on a receiver fault, whereas negative ΔCFS tends to inhibit rupture.

Earthquakes occur when the applied stress surpasses the strength of a fault. Under undrained rock conditions, the change in Coulomb Failure Stress (ΔCFS) is evaluated using the Coulomb failure criterion, which quantifies proximity to failure and is expressed as follows [48]:

(6) $$\Delta CFS=\Delta \tau +\mu^{\prime }\Delta \sigma$$

where Δτ and Δσ are the static shear stress changes and the product of effective normal stress changes, respectively, while μ’ is the coefficient of friction [49]. Moreover, Δτ is positive for increasing shear stress on the observing fault in the direction of relative slip, while Δσ is positive for increasing tensional normal stress. Failure is encouraged if ΔCFS is positive and it is discouraged if negative.

ΔCFS values were computed using Coulomb 3.3 software [50] within an elastic half-space. Positive ΔCFS, often referred to as stress bright zones, brings a fault closer to failure and increases its likelihood of rupture, whereas negative ΔCFS, or stress shadows [51,52], moves it further from failure. Accordingly, blue regions indicate negative Coulomb stress changes and reduced rupture potential, while yellow-to-red regions denote positive changes and an elevated likelihood of rupture.

**Figure 8:**
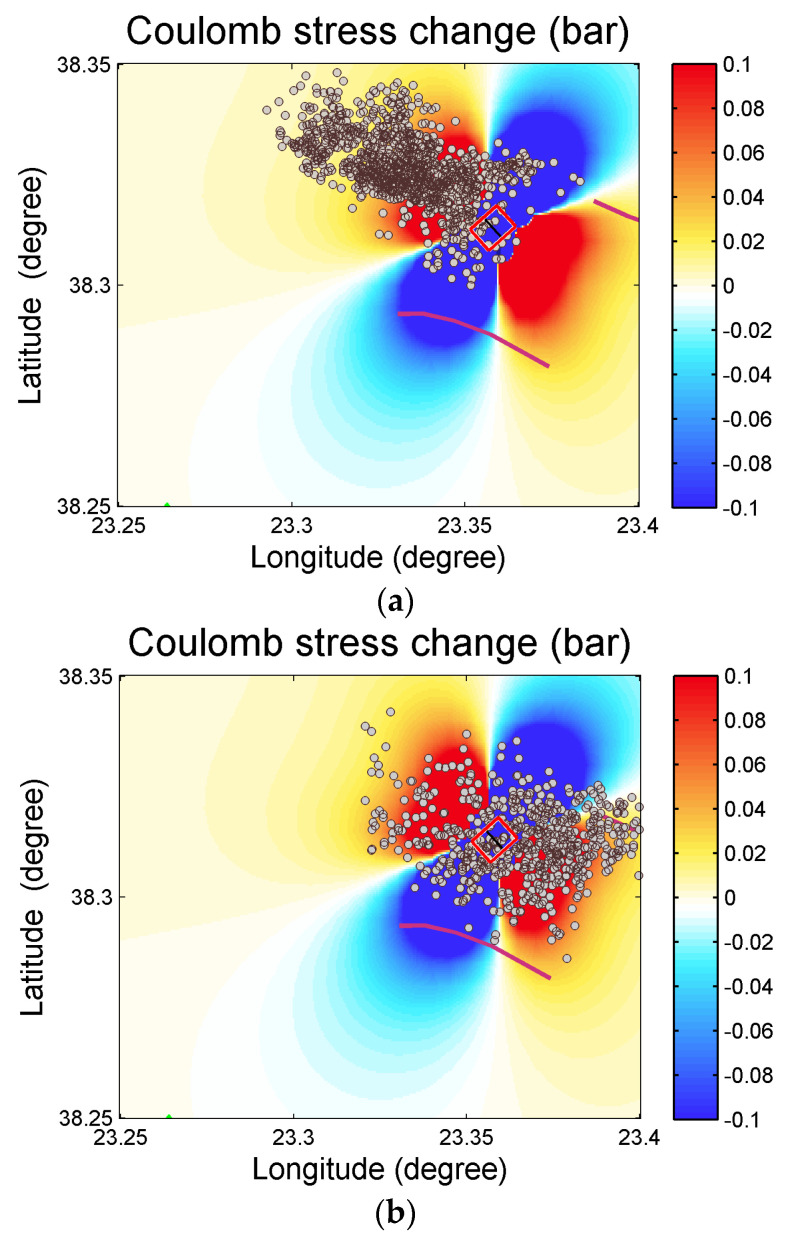
Coulomb stress changes produced by the *M*_w_ 4.0 earthquake on 2 September 2021 at a centroid depth of 7 km. The red rectangle shows the fault model for the kinematics of the *M*_w_ 4.0 event. The solid purple lines show the area faults [7], while the brown circles are the epicenters of (**a**) Cluster 2 and (**b**) Cluster 5.

The calculated co-seismic result for the ΔCFS changes for the seismic event on 2 September 2021 of magnitude *M_w_* 4.0 is presented in Figure 8. The focal parameters that were used for the estimation of ΔCFS are as follows: depth of 7 km, strike of 318°, dip of 33°, and rake of −61°, according to the moment tensor solutions of the Institute of Geodynamics, National Observatory of Athens “http://bbnet.gein.noa.gr/ (accessed on 2 July 2025)”. The spatial distribution of the ΔCFS reveals a stress decrease towards NE and WS and a stress increase towards NW and SE, coinciding with the location of Clusters 2 and 5 (Figure 8). Notably, the *M_w_* = 4.0 event appears to have played a key role in triggering Cluster 2, as the majority of subsequent earthquakes occurred in regions of positive Coulomb stress changes. This stress transfer brought adjacent fault segments closer to failure, promoting seismic activity. A similar pattern is observed in Cluster 5, which initiated shortly after the *M_w_* = 4.0 event, further supporting the hypothesis of stress-driven activation. The occurrence of earthquakes in areas of increased ΔCFS motivated the investigation of aseismic processes such as afterslip and stress diffusion. We note that the above result is in agreement with that reported in [53] for the major earthquakes that occurred on 2 December 2020, where the same orientation of the positive Coulomb stress changes was observed, suggesting a stable stress transfer direction throughout the sequence.

#### 3.3.2. Evidence of Afterslip Migration of Seismicity in Cluster 2

The spatiotemporal distribution of a swarm seismic sequence is determined by static and dynamic stresses [54] or by fluid diffusion [55]. Here, we examined the expansion of Thiva’s Cluster 2 swarm over time. Perfettini et al. [56] proposed an analytical model predicting key features of this migration along strike, attributed to co-seismic deformation. According to their model, aftershock activity is governed by afterslip loading asperities, which in turn controls the seismicity rate. In this approach a fault is modeled with depth-dependent variations in normal stress, stressing rate, and the introduction of a rheological parameter A [45,56,57,58,59].

Following this approach (see details in [56,59]), the average migration of the aftershock zone becomes:

(7) $$<\Delta L_{a}\left(t\right)>=\;<L_{a}\left(t\right)>-{<L}_{a}\left(t_{1}\right)>=\;\zeta A\frac{R}{\Delta \sigma }\ln\left(\frac{t}{t_{1}}\right)$$

where $<\Delta L_{a}\left(t\right)>$ is the mean migration distance between times $t_{1}$ and t; ζ is a constant parameter (with a value close to 2.77), which scales the average gradient of the Coulomb stress along the strike; the radius of the coseismic rupture is *R*; and the mean coseismic stress drop is Δ*σ*.

**Figure 9:**
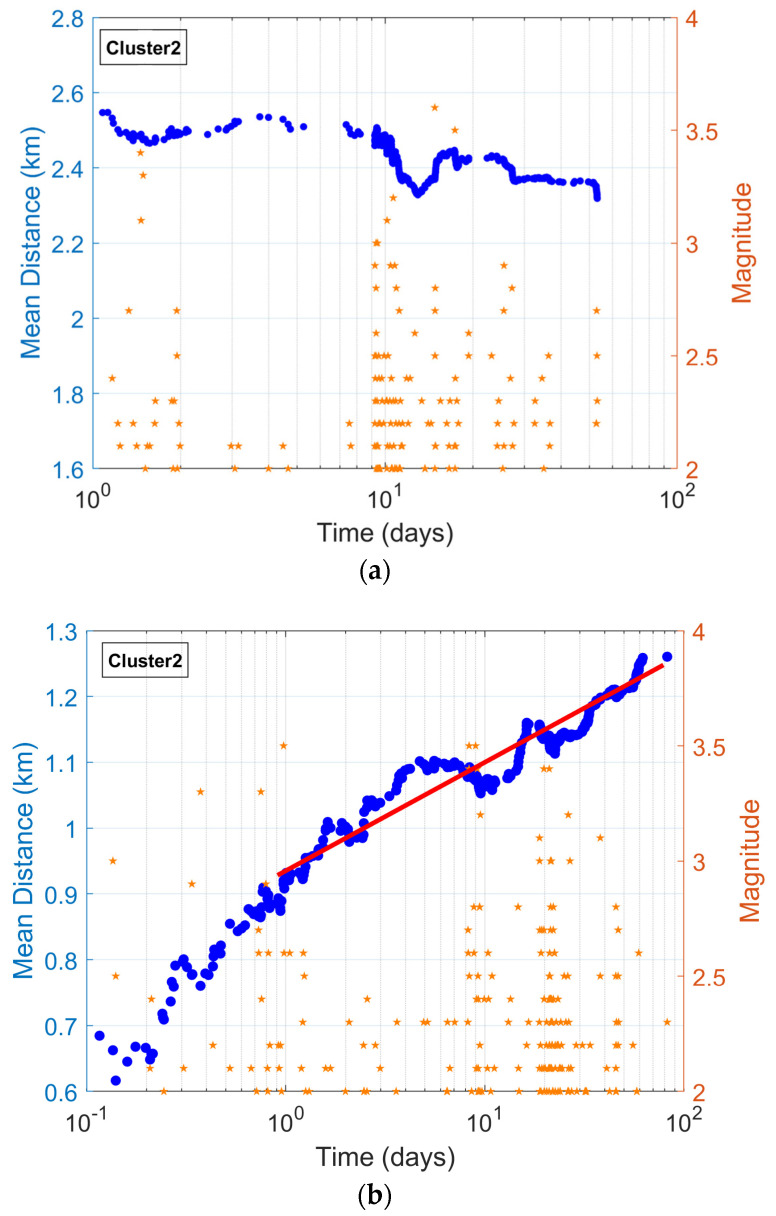
(**a**) The mean migration distance (blue circles in figures) as a function of the logarithm of time for Cluster 2 of Thiva, after the 7 July 2021, *M* 4.3 event. (**b**) The mean migration distance as a function of the logarithm of time for Cluster 2 of Thiva, after the 2 September 2021, *M* 4.0 earthquake. The orange stars illustrate the seismic events of magnitude over 2, while the red line in (**b**) presents the logarithmic trend in the evolution of mean migration distance.

Figure 9a shows the migration of seismicity following the 7 July 2021, *M* 4.3 event. It is obvious that Equation (7) does not describe the mean migration distance observed. In contrast, ten days after the *M* 4.3 event, a decrease in $<\Delta L_{a}\left(t\right)>$ was characterized by the mean migration distance, which is associated with an increase in microseismic activity; possibly, a forerunner indicated the preparation of a forthcoming event on 2 September 2021, with a similar size (*M* 4.0). Note that a similar observation is reported for the Cefalonia Island event in [59].

After the 2 September 2021, magnitude *M* 4.0 earthquake, the mean migration distance is calculated (see Figure 9b). The $<\Delta L_{a}\left(t\right)>$, which occurred a day after the *M* 4.0 event, presents a logarithmic with-time migration of the seismicity in agreement with Equation (7). The latter implies that the spatiotemporal evolution of Cluster 2 following the *M_w_* = 4.0 event shows a logarithmic migration pattern over time, aligning with theoretical models of aftershock migration driven by afterslip loading asperities. This behavior indicates that the *M_w_* = 4.0 earthquake was not only an isolated rupture but also signaled the beginning of a secondary phase of the swarm governed by aseismic slip.

#### 3.3.3. Evidence of a Diffusion Process of Seismicity of Cluster 5

Among the elementary processes that underly seismic phenomena, diffusion is undoubtedly one of the most ubiquitous [60,61]. In a first approximation, diffusion is modeled by Brownian motion. In its simplest version, Brownian motion is a random walker where earthquake hypocenters move at discrete time steps and migrate (“jump”) with equal probability [60]. This stochastic process can be readily generalized, with time made continuous by introducing random waiting times between jumps. Finally, the length of each jump can also be chosen at random from a continuous set with a prescribed probability distribution [61].

A typical solution to the Brownian diffusion problem shows that the width of the spatial distribution grows with time as follows:

(8) $$<r^{2}>\sim Dt$$

where *D* is the normal diffusion coefficient [60,61].

This proportionality between mean square distance and time is the fingerprint of normal diffusion, as it can be used to detect this kind of transport mechanism in a given natural process. Despite the fact that diffusion is a well-accepted transport mechanism in natural processes, it is known that at the mesoscopic level diffusion-like mechanisms, which violate Equation (8), exist and are generally referred to as anomalous diffusion [61,62]. More specifically, anomalous diffusion has been devoted to processes where, for large t, the mean square distance varies with time as follows:

(9) $$<r^{2}>\;\sim t^{\alpha }$$

where *α* is the dynamic exponent, or random walk fractal dimension, of the transport process [62].

To quantify the rate of earthquake diffusion, we used notions of the random walk theory to estimate the mean squared distance $\langle r^{2}\left(t\right)\rangle$ of seismicity with time. The 3D Euclidean distance r of each event is calculated from the reference point of the swarm and the estimated mean squared distance as follows [63,64,65]:

(10) $$\langle r^{2}\left(t\right)\rangle =\frac{1}{N(t)}\;\sum_{n=1}^{N(t)}r_{n}^{2}\left(t\right)\;\;\;\;\;\;\;\;$$

where $r_{n}(t)$ is the distance of the nth event at time *t* from the origin and *N* is the total number of events.

As mentioned, in normal diffusion (Gaussian), the mean squared distance linearly depends on time. However, in complex heterogeneous media, other functions are encountered based on Equation (9) that take the form of a power law [62,66]:

(11) $$\langle r^{2}(t)\rangle =D_{a}t^{a}$$

where *D*_a_ is a generalized diffusion coefficient that denotes the instantaneous response of seismicity to the diffusive process, while the diffusion exponent *α* can provide a quantitative measure of the rate of the triggered earthquake diffusion [67]. Normal diffusion corresponds to α = 1. For α < 1, the growth rate of the mean square displacement is smaller than in normal diffusion, and transport is consequently said to be subdiffusive. On the other hand, for α > 1, the mean square displacement grows relatively faster, and transport is superdiffusive [67,68,69,70].

**Figure 10:**
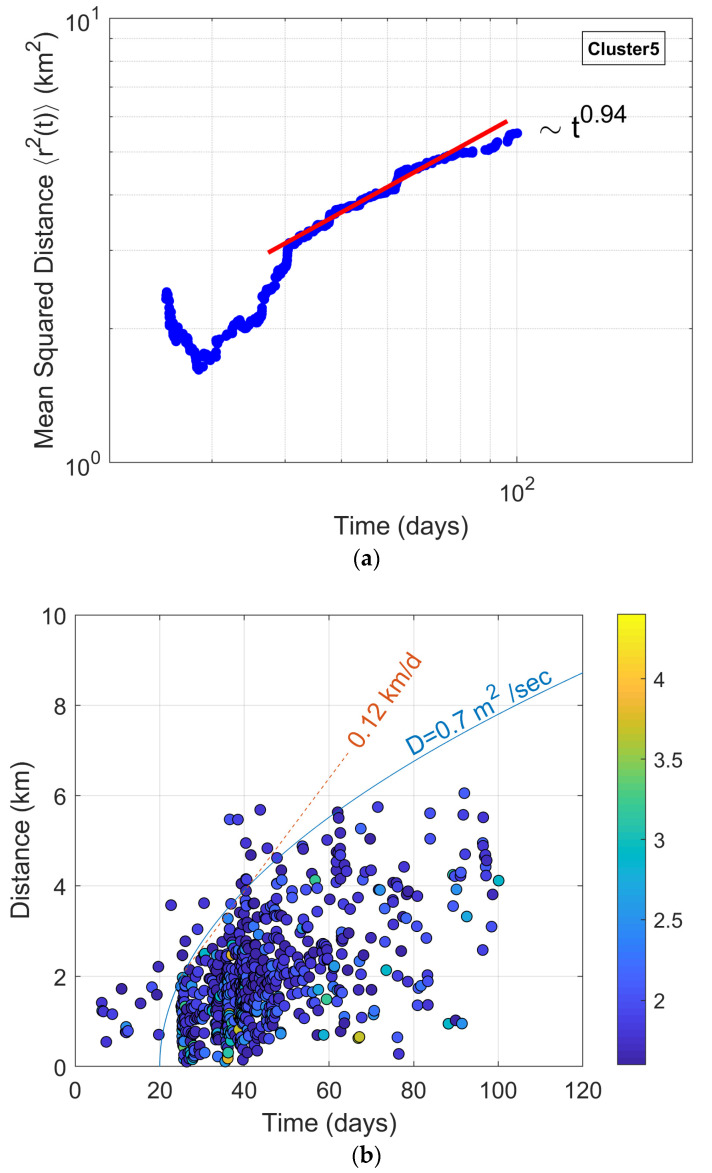
(**a**) Mean square distance 〈r^2^(*t*)〉 of seismicity (blue circles) with time t in double logarithmic axes, for Cluster 5 of Thiva. The red line indicates the power-law growth of 〈r^2^(*t*)〉 according to Equation (11). (**b**) Migration of Thiva’s Cluster 5 (see details in text). The events are colored according to magnitude. The solid blue line represents the parabolic triggering front with *D* = 0.7 m^2^/s, and the average initial seismicity migration speed, presented in km/day, is drawn by a dashed orange line.

Taking as a reference point the mean position of the first 10 events of Cluster 5, the mean squared distance with time was estimated using Equation (10). The result is shown in Figure 10a in double-logarithmic axes. The mesoscopic scaling behavior can be approximated, for large t, with the power-law relation of Equation (11) (see Figure 10a), yielding the parameter value of α = 0.94, with a correlation coefficient of R^2^ = 0.92. The diffusion exponent α being quite close to 1 suggests that the swarm migration is approximately normal (Gaussian) diffusion in nature. This near-linear growth of mean square distance with time indicates that stress diffusion dominates the process, with only slight deviations from ideal diffusion behavior [63].

The observed diffusion process is usually associated with pressurized fluid migration that could generate earthquakes in the volume behind a triggering front that spreads through the fracture network, increasing pore pressure and reducing the effective normal stress on a fault plane, allowing slip to occur [71]. Within a normal diffusion mechanism, the expanding spherical triggering front of induced seismicity, in an isotropic and homogeneous medium, can be modeled with a parabolic envelope that is characteristic for pore-fluid pressure diffusion, according to the following expression [71,72,73]:

(12) $$r(t)=\sqrt{4\pi Dt}$$

where *r*(*t*) is the radius of the propagating triggering front (which is a sphere in a homogenous isotropic medium) caused by pore-fluid pressure diffusion, *t* is the time since the beginning of the pore-pressure perturbation, and *D* is the hydraulic diffusivity.

Equation (12) can be considered as an equation for the earthquake triggering by a fluid-related mechanism of earthquake sequences in tectonic environments [73,74].

The diagram in Figure 10b indicates that as seismicity evolved during the first days (from 3 March 2022), the events systematically migrated away from the origin, forming a seismic front with an average initial velocity of 0.12 km/day. In addition, as can be observed, most of the migrating seismicity is located behind the parabolic triggering front envelope of hydraulic diffusivity *D* = 0.7 m^2^/s, although some sparse events are located on the outside, possibly triggered due to pore-fluid diffusion following paths of higher hydraulic diffusivity values [53,75,76].

The evolution of the earthquake activity follows the pore-fluid pressure triggering front with hydraulic diffusivity values ranging from 0.01 to 10 m^2^/s within the Earth’s crust and usually less than 1 m^2^/s [75,76,77,78,79,80]. The seismic front, however, may also be described with a linear triggering front with a constant velocity of the order of 0.1 to 1 km/h. Such migration velocities are consistent with a fluid-related triggering mechanism [75], while faster velocities have been associated with an aseismic slip driving force [77,78,79,80].

At this point, we clarify that both clusters were analyzed using both models. Cluster 2 did not provide evidence for a normal diffusion mechanism, while Cluster 5 did not produce meaningful results for an afterslip mechanism.

## 4. Discussion

The 2020–2022 seismic sequence at Thiva includes the mainshock and aftershock sequence in December 2020 at the Kallithea fault, as well as a seismic swarm from July 2021 to June 2022 near the city of Thiva. The seismic activity began on 10 July 2021, in the western part of the swarm, with subsequent seismic events demonstrating a general trend of spatiotemporal migration towards the east–southeast. The final significant event occurred on 10 April 2022, with a magnitude of *M*_w_ 4.4. Consequently, the seismic swarm can be divided into two parts: the western and the eastern clusters (Figure 4c).

To study spatiotemporal scaling properties and seismic energy distributions, we separated the swarm into clusters using the Density-Based Spatial Clustering of Application with Noise (DBSCAN). Specifically, we applied this algorithm to the relocated seismic events of the Thiva earthquake swarm with *M* ≥ *M*_c_ and identified five clusters (two main and three smaller clusters) based on space and time. In each main cluster of the Thiva sequence, the ideas of Non-Extensive Statistical Physics (NESP) are applied to the inter-event times and distances as well as the magnitude distributions.

In a number of previous works, it has been demonstrated that the spatiotemporal distribution of seismicity as expressed by the interevent distances [35] and the interevent times [33] distributions follows a *q*-exponential distribution with *q_D_* < 1 and *q_T_* > 1, respectively. In our study, the non-additive entropic parameters range from 0.7 to 0.8 for inter-event distances, whereas the corresponding ones for inter-event times range between 1.44 and 1.5. These *q*-values are in agreement with the *q*-values found in other studies (see [33,35,53,57,81,82] and references therein).

The entropic parameter estimates strongly suggest that in each cluster of the earthquake swarm, the temporal evolution is described by low degrees of freedom, indicating a high level of organization, hierarchy, and non-additive characteristics in agreement with the results presented in [37].

The spatiotemporal distribution of a swarm seismic sequence is determined by static and dynamic stresses or by fluid diffusion. Here, the expansion of Thiva’s Cluster 2 swarm over time was studied. Following [56,58], aftershock activity is governed by afterslip loading asperities, which in turn controls the seismicity rate. In this approach, a fault is modeled with depth-dependent variations in normal stress, stress rate, and the introduction of rheological parameter A, leading to a logarithmic with-time evolution of migration. In Cluster 2, the major earthquakes of the swarm in July 2021 occurred at its western end, and the swarm progressed eastward. There is no significant migration of seismicity after the 7 July 2021, *M* 4.3 event. In contrast, ten days after the *M* 4.3 event, a decrease in mean migration distance was observed associated with the increase in microseismic activity, possibly indicating the accumulation of stress and the preparation of the forthcoming event of 9 September 2021, with a similar size (*M* 4.0). Following a short interval of low seismic activity in the second half of August 2021, a second stage of the swarm was initiated in September 2021. After the 9 September 2021, *M* 4.0 earthquake, the mean migration distance presents a logarithmic with time migration of the seismicity in agreement with a mechanism of aftershock migration driven by afterslip [56].

Cluster 5, which is located in the east–southeast part of the swarm and starts in March 2022, exhibits diffusion characteristics. The mean squared distance of epicenters scales with time, with a diffusion exponent α being quite close to 1, implying that the swarm migration is approximately normal (Gaussian) diffusion in nature. Furthermore, seismicity evolved during the first days (from 3 March 2022), with events systematically migrating away in the SSE direction with an average initial velocity of 0.12 km/day, presenting a hydraulic diffusivity of *D* = 0.7 m^2^/s. The intrusion of fluids through the fracture network can induce seismicity either by localizing stress or by increasing pore pressure, reducing the effective normal stress, and facilitating aseismic creep. The evolution of seismicity in Cluster 5, which seems to be fluid-driven, helps small asperities in the fractured zone to break, transferring static stress to neighboring fault patches where events are triggered in agreement with that presented in [53].

## 5. Concluding Remarks

In this work, using a relocated catalog of seismicity, we study the physical and statistical pattern of the Thiva swarm activated after the 2 December 2020 earthquake with a magnitude of *M*_w_ 4.5 that occurred near the city of Thiva. The swarm displayed a general trend of spatiotemporal migration toward the east–southeast until the middle of 2022.

The DBSCAN clustering algorithm was subsequently applied to the 2020–2022 Thiva earthquake sequence to identify spatiotemporal earthquake clusters. The scaling properties of the main clusters were analyzed using the Non-Extensive Statistical Physics (NESP) approach, providing detailed insights into the nature of the long-term correlation of the seismic swarm. The results of the analysis indicate that the statistical properties of earthquake swarm clusters can be successfully reproduced by means of NESP and confirm the complexity and non-additivity of the spatiotemporal evolution of seismicity.

The entropic parameters were estimated with *q_D_* values ranging from 0.7 to 0.8 and *q_T_* values from 1.44 to 1.50. Furthermore, the frequency–magnitude distributions were analyzed using the fragment–asperity model proposed within the NESP framework, providing the non-additive entropic parameter (*q_M_*). The results suggest that the statistical characteristics of earthquake clusters can be effectively interpreted using NESP, highlighting the complexity, hierarchy, and non-additive nature of the spatiotemporal evolution of seismicity in agreement with a system described by low degrees of freedom.

In addition, possible physical mechanisms responsible for the evolution of the two main and larger clusters are suggested. For Cluster 2, located in the west–northwest part of the activated zone, an afterslip mechanism activated after the 2 September 2021, *M* 4.0 events seems to control its evolution, while for the second cluster located in the east—south part of the fractured zone (Cluster 5), a normal diffusion mechanism is proposed to describe its migration pattern. Subsurface fluids under pressure seem to have played a significant role in the evolution of the Cluster 5, 2021–2022 seismic swarm, either causing local stress concentrations or facilitating slip by lowering the effective normal stresses or weakening the faults’ walls through erosion.

## Figures and Tables

**Table 1:** Clusters identified, their duration, and the number of events classified using the DBSCAN algorithm in the Thiva earthquake swarm catalog.

Clusters	Period Time	Events
Cluster 1	2 December2020–15 December 2020	10
Cluster 2	1 July 2021–23 November 2021	1018
Cluster 3	28 January 2022–1 March 2022	32
Cluster 4	1 February 2022–21 February 2022	9
Cluster 5	2 March 2022–10 June 2022	644

**Table 2:** The estimated entropic parameter *q_M_* for each cluster (for details, see text and [11]).

Clusters	*q_M_*
Unified Cluster	1.65 ± 0.01
Cluster 2	1.63 ± 0.02
Cluster 5	1.68 ± 0.02

**Table 3:** The estimated entropic parameters *q_T_* and *q_D_*, and the generalized scaled interevent time–distance, *T_q_* and *D_q_*, (in analogy with X_0_ in Equation (4)) along with their 95% confidence intervals.

Clusters	*q_T_*	*Tq* (s)	*q_D_*	*Dq* (km)
Unified Cluster	1.50	1380 ± 4	0.70	2.00 ± 0.01
Cluster 2	1.50	1095 ± 3	0.80	1.45 ± 0.01
Cluster 5	1.44	2210 ± 13	0.78	2.45 ± 0.02

## Data Availability

Data are openly available at the Seismological Laboratory of the National and Kapodistrian University of Athens (SL-NKUA, http://www.geophysics.geol.uoa.gr/) for Thiva (last accessed on 2 July 2025). Seismological waveform data from permanent and temporary stations of the HUSN are available at the European Integrated Data Archive (EIDA) node hosted at GI-NOA (http://eida.gein.noa.gr/; accessed on 2 July 2025).
